# Ixazomib combined with lenalidomide and dexamethasone chemotherapy for newly diagnosed multiple myeloma in China—Compared with bortezomib/lenalidomide/dexamethasone

**DOI:** 10.1002/cam4.5198

**Published:** 2022-09-02

**Authors:** Zhongjun Huo, Fang Chen, Ping Liu, Zimian Luo

**Affiliations:** ^1^ Department of Hematology Central Hospital of Xiangtan XiangTan China

**Keywords:** adverse events, bortezomib, Ixazomib, newly diagnosed multiple myeloma, real world

## Abstract

**Background:**

To compare the response and safety of Ixazomib/Lenalidomide/Dexamethasone (IRd) and Bortezomib/Lenalidomide/Dexamethasone (VRd) treatment in newly diagnosed multiple myeloma (MM).

**Methods:**

This was a single‐center retrospective analysis in Xiangtan Central Hospital. A total of 52 newly diagnosed MM patients from June 2019 to June 2021 were enrolled and divided into the IRd (*n* = 21) and VRd (*n* = 31) groups. After 4 cycles of chemotherapy, the best response and adverse events were recorded. Moreover, the progression‐free survival (PFS) and overall survival (OS) were calculated.

**Results:**

Patients in IRd group and VRd group showed similar PFS (Log‐rank *p* = 0.70), OS (Log‐rank *p* = 0.61) and overall response rate (83.87% vs 90.48%, *p* = 0.803). In addition, patients in VRd group showed lower Eastern Cooperative Oncology Group scores (*p* = 0.047), and higher incidence of peripheral sensory neuropathy (0.00% vs 19.35%, *p* = 0.032) than that of patients in IRd group.

**Conclusion:**

Compared to VRd regimen, IRd had the similar efficacy, better safety, and may be more convenient for patients with poor basic condition for newly diagnosed MM. This study provides an insight for physicians to use IRd as first‐line treatment in MM.

## Graphical abstract

This study indicats the similar efficacy, PFS, and OS in patients treated by VRd and IRd. Moreover, IRd shows better safety than VRd, and it may be more convenient for patients with poor basic condition. This study provides an insight for physicians to use IRd as first‐line treatment in MM.

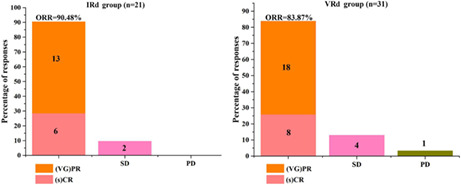

## 1 INTRODUCTION

Multiple myeloma (MM) is a type of incurable clonal plasma cell disease that most frequently occurs in the elderly.^1^ Epidemiology investigation by International Agency for Research on Cancer showed that, only in 2020, the new diagnosed and death cases of MM worldwide were approximately 176,400 and 11,7000, respectively, ranking third in all hematological malignancies.^2^ The traditional therapeutic approach for MM is chemotherapy or chemotherapy combination with stem cell transplantation. In recent years, with the more uniform use of autologous stem cell transplantation and remarkable advance in developing novel agents, such as proteasome inhibitor, monoclonal antibody, and immunomodulator, the overall survival (OS) of MM has been obviously improved.^3^, ^4^, ^5^, ^6^ However, its 10‐year survival ratio remains at only 17%.^7^ Therefore, it is imperative to develop new therapy regimens to improve prognosis.

Bortezomib, the first generation of proteasome inhibitor, is the first‐line agent of MM recommended by the National Comprehensive Cancer Network guidelines,^8^ which distinctly improved the curative effect and survival time of MM patients. Despite being one of the most widely utilized and highly effective drugs for MM, bortezomib triggers untoward effects, such as peripheral neuropathy, skeletal muscle and cardiac, as well as myelosuppression, that possibly result in termination of treatment in advance with negative outcome of patients,^9^ emphasizing the necessity to develop safer and more effective alternatives. Regarded as a second‐line recommended agent, ixazomib is a second‐generation proteasome inhibitor and shows excellent therapeutic impact in patients with relapsed/refractory MM.^10^ But the efficacy and safety of ixazomib as the first‐line treatment for MM patients in China has been barely reported and compared.

In this work, we retrospectively compared the efficacy and safety of Ixazomib/Lenalidomide/Dexamethasone (IRd) and Bortezomib/Lenalidomide/Dexamethasone (VRd) on newly diagnosed MM patients.

## 2 MATERIALS AND METHODS

### 2.1 Patients

Eligible newly diagnosed MM patients were confirmed according to the criteria of International Myeloma Working Group (IMWG). Patients in combination with other hematological disease, malignant tumor, or with an expected survival of less than 3 months and chemotherapy using current regimens for less than 4 cycles were excluded. All the enrolled MM patients provided informed consent.

### 2.2 Study design

This was a retrospective, single‐center study in Xiangtan Central Hospital from June 2019 to June 2021, and approved by the Medical Ethics Committee of Xiangtan Central Hospital (Approval number: 2019–01‐007). A total of 52 newly diagnosed MM patients were enrolled and divided into the IRd (*n* = 21) and VRd (*n* = 31) groups. The collected data of patients included gender, age, International Staging System (ISS) stage, Eastern Cooperative Oncology Group (ECOG) performance status, results of blood test (Blood routine examination, renal and liver function, serum immunofixation electrophoresis, M protein, serum protein electrophoresis, serum free light chain, β2‐microglobulin (β2‐MG) concentration, lactic dehydrogenase (LDH)), urine test (Urine routine examination, urine immunofixation electrophoresis, urine Bence‐Jone protein, and urine free light chain), imaging modality (X‐ray, low‐dose whole‐body computed tomography [CT], positron emission tomography‐CT), and bone marrow examination (Bone marrow cytology, flow cytometry immunophenotyping, cytogenetics).

VRd is considered as the standard first‐line therapeutic strategy for newly diagnosed MM. IRd is an oral regimen, which is convenient for elderly patients with poor basic condition, especially in the case of COVID‐19. IRd or VRd regimen is recommended according to the comprehensive evaluation of the patient's condition, and patients make informed choices. Patients in IRd group received oral ixazomib at the dose of 3 mg or 4 mg (dose selected according to the kidney and liver state of patients) on the days of 1, 8, and 15, lenalidomide at the dose of 10 mg or 20 mg (dose regulated depending on the creatinine clearance rate) on 1–21 days, dexamethasone at the dose of 20–40 mg (dose regulated according to the age) on the days of 1, 8, 15, and 22 with one cycle of 28 days. Patients in VRd group received subcutaneous injection of bortezomib at dose of 1.3 mg/m^2^ on days of 1, 4, 8, and 11, combined with oral lenalidomide at the dose of 10 mg or 20 mg (dose regulated depending on the creatinine clearance rate) on 1–21 days and dexamethasone at the dose of 20–40 mg (dose regulated according to the age) on days of 1, 8, 15, and 22, with one cycle of 28 days. After treatment the best response, adverse events, and concentration of hemoglobin (HGB), β2‐MG, white blood cells (WBC), platelets (PLT), Creatinine (CRE), LDH, globulin (GLB), and serum calcium (Ca) were recorded.

### 2.3 Outcomes and disease assessments

The primary endpoint was overall response rate (ORR), and the secondary endpoints included progression‐free survival (PFS), overall survival (OS), and adverse events incidence.

Treatment efficiency was evaluated according to the IMWG criteria (2016). Determining the concentration of immune globulin before each cycle of therapy, combined with other examination such as marrow cell, bone marrow flow cytometry immunophenotyping, serum and urine light chain, M protein, serum protein electrophoresis, and serum and urine immunofixation electrophoresis after 4 cycles of treatment. Thereafter, the best responses of each patient were recorded, including complete remission (CR), stringent complete response (sCR), partial response (PR), very good partial response (VGPR), stable disease (SD), progressive disease (PD). The overall response rate (ORR) was calculated using formula as follow: (SUM number of CR, sCR, PR, VGPR)/cases total number × 100%.

PFS was calculated from diagnosis to progression of disease, death, or the last follow‐up, whereas OS was calculated from diagnosis to death or the last follow‐up.

Adverse events were graded according to the data derived from the Common Terminology Criteria for Adverse Events.

### 2.4 Statistical analysis

Statistical analysis was performed using SPSS, version 20.0 (SPSS Inc., Chicago, IL, USA). Enumeration data were represented as frequency and percentage (%), and texted using x^2^ and rank sum test. Measurement data were expressed as mean ± standard deviation (*SD)*, and tested using Student's *t*‐test. OS and PFS were analyzed using GraphPad Prism, version 7.00 (GraphPad Software Inc., La Jolla, CA, USA), and statistical analysis was performed using the Kaplan–Meier method followed by the log‐rank test. *p* < 0.05 was considered to be statistically significant.

## 3 RESULTS

### 3.1 Baseline characteristics

There were 8 male and 13 female patients in IRd group with a mean age of 69‐year‐old (range, 40–86). Of them, 8 patients had high‐risk cytogenetics, 8 had standard‐risk cytogenetics, the remaining 6 patients did not undergo cytogenetic risk stratification due to the lack of fluorescence in situ hybridization. Whereas, among 31 patients in VRd group, 15 were male and 16 were female with a mean age of 67 (range, 53–83). Of them, 12 patients had high‐risk cytogenetics, 11 had standard‐risk cytogenetics, the remaining 8 patients did not undergo cytogenetics risk stratification due to the lack of fluorescence in situ hybridization. In addition, 13 patients in the IRd group and 19 patients in VRd showed bone lesion. The clinical characteristics of MM patients were shown in Table 1. Compared to the patients in VRd group, the gender distribution, age of diagnosis, HGB concentration, β2‐MG concentration, ISS stage, creatinine clearance, and cytogenetics risk stratification of patients in IRd group demonstrated no significant statistical difference (*p* > 0.05). However, there was significant statistical difference in ECOG scores between IRd group and VRd group at initial diagnosis (*p* = 0.047) (Table 1), indicating that the general basic condition of patients in the IRd group was significantly worse than that in the VRd group.

**TABLE 1:** Baseline characteristics of the patients in two groups

Characteristics	IRd group (21)	VRd group (31)	*p*‐value
Gender, Male/Female, number	8/13	15/16	0.473
Mean age, year (range)	69.14 (40–86)	66.87 (53–83)	0.400
Mean HGB, g/l (range)	85.20(50–123)	88.50 (44–146)	0.741
Mean β2‐MG, μg/ml (range)	8.91 (2.54–19.95)	6.31 (1.15–25.20)	0.186
Bone lesion, number (%)	13 (61.90)	19 (61.29)	0.965
ECOG, number (%)			
1	5 (23.81)	13 (41.94)	
2	7 (33.33)	14 (45.16)	0.047
3	9 (42.86)	4 (12.90)	
ISS stage, number (%)			
I	3 (14.29)	6 (19.35)	
II	9 (42.86)	13 (41.94)	0.886
III	9 (42.86)	12 (38.71)	
Creatinine clearance, number (%)			
X<30 ml/min	5 (23.81)	5 (16.13)	
30<X<60 ml/min	6 (28.57)	7 (22.58)	0.612
X ≥ 60 ml/min	10 (47.62)	19 (61.29)	
Cytogenetic risk stratification, number (%)			
Standard risk	7 (33.33)	12 (38.71)	
High risk	8 (38.10)	11 (35.48)	0.924
Not available	6 (28.57)	8 (25.81)	

Abbreviations: β2‐MG, β2‐microglobulin concentration; HGB, hemoglobin concentration.

### 3.2 Response assessment

**FIGURE 1:**
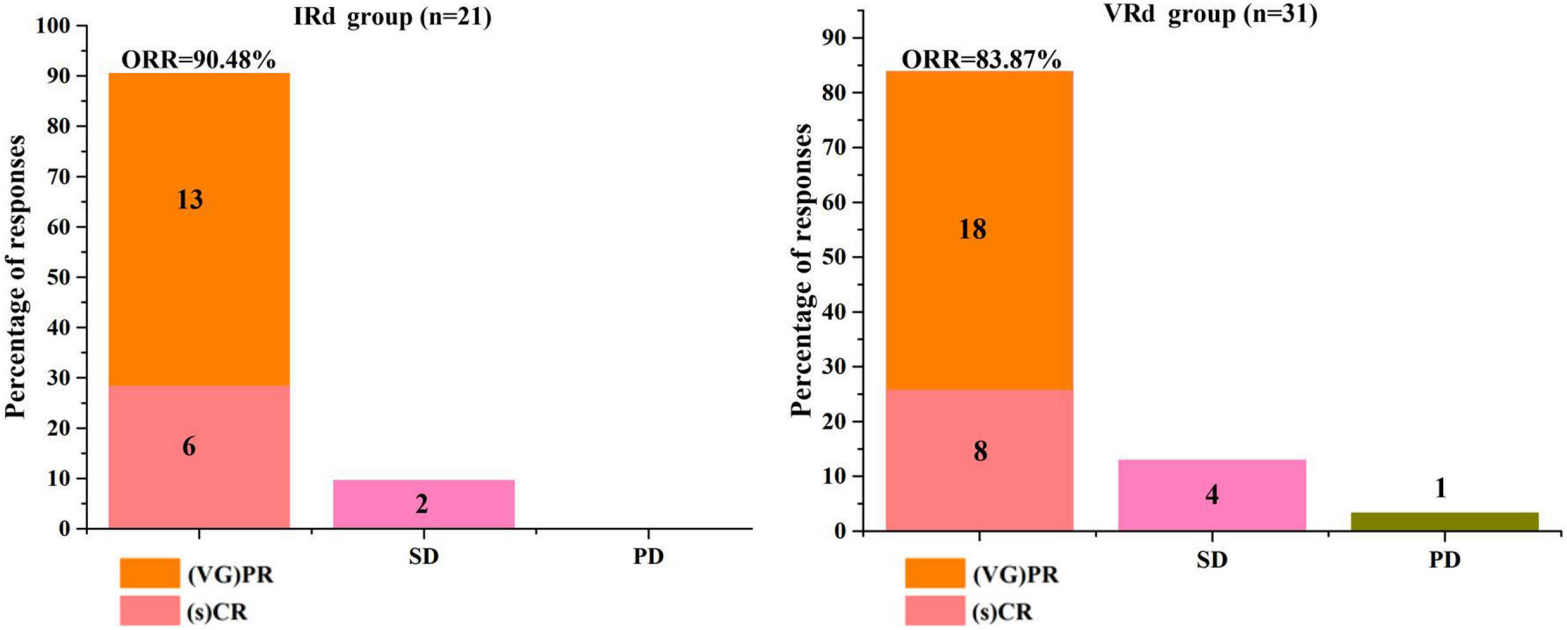
Best response of patients in IRd and VRd group. CR, complete response; ORR, overall response rate; PD, progressive disease; sCR, stringent complete response; (s)CR, sCR plus CR; SD, stable disease; (VG)PR, VGPR plus PR; VGPR, very good partial response.

Blood tests found that both IRd and VRd treatment increased the concentration of HGB, LDH, and decreased the concentration of WBC, PLT, β2‐MG, CRE, and GLB. Although, some of them showed no significant statistical difference (Table 2). Among 21 patients in IRd group, 4 patients (4/21, 19.04%) achieved CR, 2 patients (2/21, 9.52%) achieved sCR, 4 patients (4/21, 19.04%) achieved VGRP, and 9 patients (9/21, 42.86%) achieved PR. Among 31 patients in VRd group, 5 patients (5/31, 16.13%) achieved CR, 3 patients (3/31, 9.68%) achieved sCR, 5 patients (5/31, 16.13%) achieved VGRP, and 13 patients (13/31, 41.94%) achieved PR. ORR were 90.48% (19/21) in IRd group, and 83.87% (26/31) in VRd group. SD was observed in 2 patients (2/21, 9.52%) in IRd group, and 4 patients (4/31, 12.90%) in VRd group (Table 3, Figure 1). PD was observed in 1 patient (1/31, 3.23%) in VRd group. Compared to VRd group, all of the corresponding responses showed no statistic difference (all *p* > 0.05) (Table 3).

**TABLE 2:** The changes of correlation indecators before and after chemotherapy of patients in the two groups

	IRd group			VRd group		
Indicator	Preliminary diagnosis	Two course	Four course	Preliminary diagnosis	Two course	Four course
HGB (g/l)	85.50 ± 24.07	96.83 ± 6.59	102.83 ± 17.99	74.71 ± 22.13	106.86 ± 9.72	107.57 ± 29.09
WBC (10^9^/l)	8.73 ± 7.52	3.92 ± 1.33	4.24 ± 1.57	5.21 ± 2.46	4.82 ± 2.65	4.50 ± 2.26
PLT (10^9^/l)	247.67 ± 154.85	188.00 ± 67.63	169.83 ± 62.82	159.71 ± 67.75	209.43 ± 68.64	140.57 ± 61.88
β2‐MG (mg/l)	8.97 ± 6.14	4.33 ± 1.69*	3.87 ± 2.35	5.66 ± 3.48	2.24 ± 0.83*	2.67 ± 1.01*
CRE (μmol/l)	96.50 ± 47.33	82.82 ± 46.15	83.67 ± 45.71	91.86 ± 27.26	71.57 ± 15.80*	66.50 ± 10.15*
LDH (μ/l)	131.17 ± 27.78	173.50 ± 41.98	166.00 ± 49.09	145.00 ± 56.91	181.33 ± 36.68	160.43 ± 32.87
GLB (g/l)	53.92 ± 19.71	29.40 ± 11.44*	27.95 ± 12.50*	49.91 ± 29.66	19.74 ± 2.14*	24.50 ± 14.65*
Ca (mmol/l)	2.41 ± 0.43	2.18 ± 0.10	2.23 ± 0.14	2.32 ± 0.25	2.25 ± 0.08	2.43 ± 0.28

Abbreviations: β2‐MG, β2‐microglobulin; Ca, serum calcium; CRE, creatinine; GLB, globulin; HGB, hemoglobin concentration; LDH, lactate dehydrogenase; PLT, Platelets; WBC, white blood cells.

* *p* < 0.05 vs. preliminary diagnosis.

**TABLE 3:** Treatment responses of patients in the two groups (*n*, %)

Group	Cases	CR	sCR	VGPR	PR	SD	PD	ORR(%)
IRd	21	4 (19.05)	2 (9.52)	4 (19.05)	9 (42.86)	2 (9.52)	0 (0.00)	90.48
VRd	31	5 (16.13)	3 (9.68)	5 (16.13)	13 (41.94)	4 (12.90)	1 (3.23)	83.87
X^2^		0.075		0.075				0.062
P		0.785		0.785				0.803

Abbreviations: CR, complete response; ORR, overall response rate; PD, progressive disease; sCR, stringent complete response; SD, stable disease; VGPR, very good partial response.

### 3.3 PFS and OS

**FIGURE 2:**
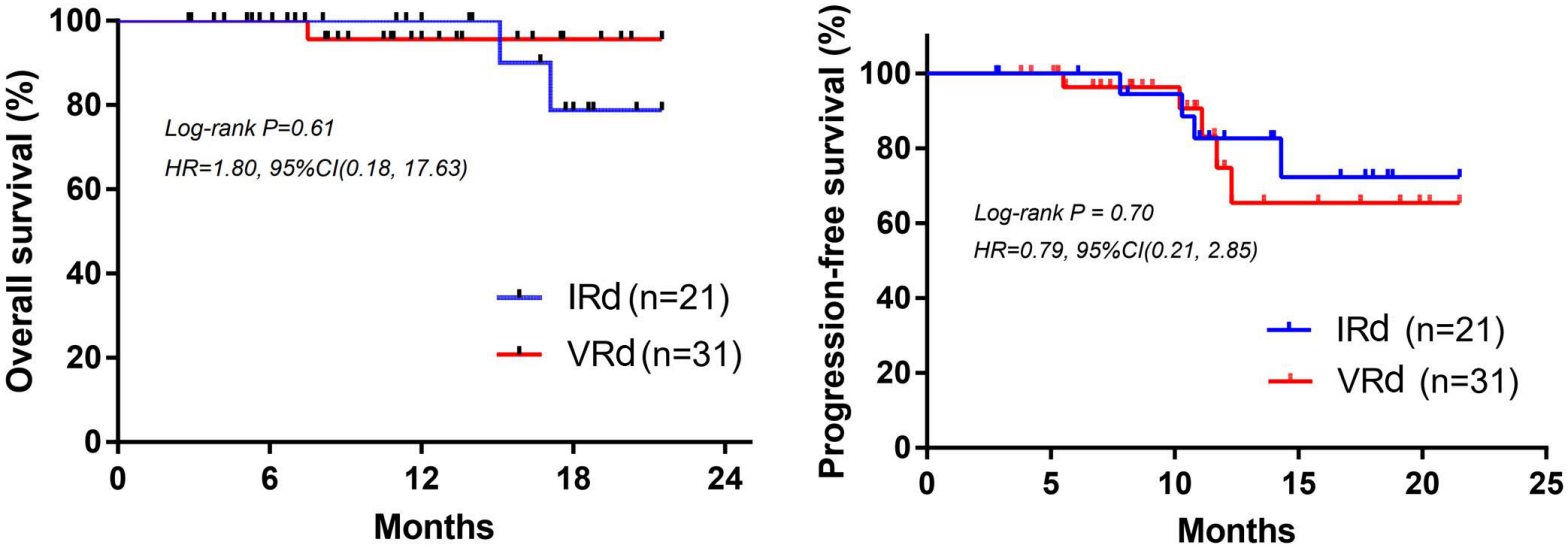
Overall survival and progression‐free survival of patients in IRd and VRd group.

The longest follow‐up was 21.5 months. Due to the short follow‐up period, the median OS and PFS were not achieved. As shown in Figure 2, there was no statistical difference of OS (Log‐rank *p* = 0.61, 95%CI, 0.18–17.63) and PFS (Log‐rank *p* = 0.70, 95%CI, 0.21–2.85) between patients in IRD and VRD groups. To better evaluate the PFS and OS in these two groups, longer follow‐up is needed.

### 3.4 Adverse events

The adverse events that occurred in patients are shown in Table 4. The main hematologic toxicities in IRd and VRd groups were leukopenia and hemocytopenia. And two patients in both IRd and VRd groups had grade 1–2 leukopenia, and 3 patients had grade 3–4 hemocytopenia in VRd group. Grade 1–2 hemocytopenia was found in 2 patients in both IRd and VRd groups. No intergroup differences were observed in the occurrence of the hematologic adverse events (all *p* > 0.05). Diarrhea, peripheral sensory neuropathy, and limb numbness were the main non‐hematologic toxicities in the patients. And two patients had diarrhea in IRd group, of which one was grade 1–2, another was grade 3–4. Six patients had peripheral sensory neuropathy in VRd group, of which 1 was grade 1–2, and 5 were grade 3–4. And three patients in VRd group had grade 1–2 limb numbness. No intergroup differences were observed in the occurrence of the Diarrhea and limb numbness. Whereas, patients who suffered from peripheral sensory neuropathy showed intergroup difference between IRd and VRd groups (0.00% vs. 19.35%, *p* = 0.032).

**TABLE 4:** Adverse events of patients in the two groups (*n*, %)

	IRd group (21)			VRd group (31)				
Toxicities	G1‐G2	G3‐G4	Total	G1‐G2	G3‐G4	Total	X^2^	P
Hematologic								
Leukopenia	2	0	2 (9.52)	2	0	2 (6.45)	0.166	0.683
Hemocytopenia	2	0	2 (9.52)	2	3	5 (16.13)	0.469	0.494
Non‐hematologic								
Diarrhea	1	1	2 (9.52)	0	0	0 (0.00)	3.071	0.080
Peripheral sensory neuropathy	0	0	0 (0.00)	1	5	6 (19.35)	4.595	0.032
Limb numbness	0	0	0 (0.00)	3	0	3 (9.68)	2.157	0.142

Abbreviations: G, Grade; IRd, Ixazomib plus Lenalidomide plus Dexamethasone; VRd, Bortezomib plus Lenalidomide plus Dexamethasone.

## 4 DISCUSSION

Proteinase is the vital constituent of the ubiquitin‐proteasome pathway, playing an important function in protein homeostasis, cellular growth, and apoptosis regulation.^11^ Bortezomib is a boronic acid‐based potent proteinase inhibitor that interferes the degradation of ubiquitinated proteins induced by 26S proteasome, thereby preferential killing a variety of tumor cells.^12^, ^13^, ^14^ Bortezomib has been initially approved as the third‐line drug for relapsed and refractory MM treatment, and subsequently, as first‐line regent for mantle cell lymphoma and MM.^15^, ^16^, ^17^ Bortezomib‐containing regimens such as VRd, bortezomib/cyclophosphamide/dexamethasone,^18^ and bortezomib/melphalan/prednisone,^19^ are considered as the standard first‐line therapeutic strategy for newly diagnosed MM. A phase II portion of the single‐arm study of VRd in newly diagnosed MM patients performed by Richardson et al. demonstrated that 100% of ORR with 74% of VGPR or better was observed after 4 cycles of treatment.^20^ Moreover, The EVOLUTION study by Kumar and colleagues revealed that an ORR of 85%, with 51% of VGPR or better was achieved in newly diagnosed MM after four cycles of VRd treatment.^21^ In our current study, after 4 cycles of VRd treatment in newly diagnosed MM patients, the ORR was 83.87% with 41.94% of VGPR or better, which was similar to that of Kumar's study. Despite the preeminent anti‐tumor activity of bortezomib, the lack of sensitivity, drug resistance, and adverse reaction to bortezomib restricted its clinical application.^22^, ^23^, ^24^

Ixazomib is a first FDA‐approved oral second‐generation proteasome inhibitor that selectively binds to the β5 subunit of the 20S proteasome and inhibits its chymotrypsin‐like activity, leading to apoptosis of multiple myeloma cells.^25^ Compared to bortezomib, ixazomib shows better advantages upon improving the administration route and decreasing toxicity.^26^ In addition, compared to bortezomib, ixazomib has shorter dissociation half‐life with the proteasome, higher dissociation rate with the proteasome and enrichment in cancer, stronger capacity to penetrate the tissues, as well as the ability to trigger apoptosis of drug‐resistant cell, therefore showing better anti‐MM activity.^26^, ^27^, ^28^ In November 2015, IRd was approved as the first oral regimens for the therapy of patients with relapsed/refractory MM.^29^ Numerous studies have demonstrated the therapeutic effect of IRd on relapsed/refractory MM.^30^, ^31^ The phase III TOURMALINE‐MM1 trial in patients with relapsed/refractory MM performed by Richardson and colleagues demonstrated that the median OS was up to 53.6 months after 18 cycles of treatment with IRd, which was the longest reported in phase III studies of IRd in relapsed/refractory MM.^32^ Few trials also investigated the efficacy and safety of IRd in newly diagnosed MM. An open‐label, dose‐escalation, phase 1/2 study of IRd in the United States newly diagnosed MM patients showed that the ORR was 88% with 32% of CR, and all the population was tolerated with IRd.^33^ Another open‐label, multicenter, phase 1/2 clinical trial in the United States newly diagnosed MM patients demonstrated that 94% of patients received ORR with 68% of VGPR or better and 24% of CR after IRd treatment.^34^ However, much more trials were needed to explore the efficacy and safety of IRd in newly diagnosed MM. Moreover, the effect of IRd in Chinese newly diagnosed MM and its efficacy verses to VRd were barely reported. In the presents study, 52 newly diagnosed MM patients in Chinses were divided into IRd (*n* = 21) and VRd (*n* = 31) groups. The patients treated with IRd received 90.48% of ORR including 28.57% of CR, which was similar to previous study.^33^, ^34^ The ORR of patients in IRd group was higher than that of the patients in VRd group (83.87%). No statistical difference existed, indicating the similar efficacy of IRd and VRd. Additionally, the PFS and OS also showed no statistical difference between patients in IRd and VRd group. The double‐blind, placebo‐controlled TOURMALINE‐MM2 trial reported by Facon et al showed that IRd treatment led to a 13.5‐month improvement in median PFS in transplant‐ineligible patients with newly diagnosed MM compared to placebo plus Rd treatment; however, no statistically significant outcome was observed and the trial did not technically meet its PFS endpoint.^35^

Previous study showed that the adverse reactions of IRd were mainly reflected in peripheral neuropathy, rashes, neutropenia, thrombocytopenia, diarrhea, and fatigue.^33^, ^34^ Nevertheless, research suggested that the drug‐related adverse reactions of IRd were mostly within the tolerable range.^33^ Clinical test demonstrated that the adverse events’ incidence of bortezomib‐associated regimes were higher than those of ixazomib‐related regimes in the therapy of relapsed/refractory MM, especially peripheral sensory neuropathy.^36^ Our current study indicated that among 21 patients in IRd group, 28.56% of patients occurred adverse reaction including 2 of grade 1–2 leukopenia, 2 of grade 1–2 hemocytopenia, 1 of grade 1–2 diarrhea and 1 of grade 3–4 diarrhea, respectively. Whereas, among 31 patients in VRd group, 51.61% of patients suffered from adverse reaction containing 2 of leukopenia, 5 of hemocytopenia, 6 of peripheral sensory neuropathy, and 3 of limb numbness. The adverse reactions incidence of VRd were much higher than those of IRd, especially peripheral sensory neuropathy.

As we know that the incidence of MM is increasing with the aging of population, and about 65% of patients diagnosed with MM are over 65 years. The elderly patients often have more basic diseases and poor physical status. In our retrospective study of 52 newly diagnosed MM, 13 patients with ECOG score of 3, accounted for 25%. These patients received low intensity of chemotherapy due to poor tolerance to adverse reactions of chemotherapy, which affected the treatment of the disease, thereby leading to disease progression and seriously reduced the quality of life of the patients. In addition, we found that the basic state of patients in IRd groups was worse than that in the VRd treatment group, as demonstrated by the higher ECOG scores of patients in IRd group. This may be associated with the way of administration, patients with poor condition prefer to choose the oral regimen (IRd), which is more convenient. In addition, oral regimen has more advantage under the condition of COVID‐19 that patients can take medication at home, which also relieves the pressure on doctors to some extent. The TOURMALINE‐MM2 trial reported by Thierry et al. also demonstrated that IRd is a feasible strategy for certain transplant‐ineligible in newly diagnosed MM patients who often have limited mobility and reduced ability to frequently attend hospital.^35^

A limitation of the current study was that this was a retrospective, single‐center study and the sample size was too small to obtain the accurate conclusions. In addition, the short duration of follow‐up may confuse the conclusion of prognosis. Collectively, the present data demonstrate the efficacy and safety of the oral regimen IRd for newly diagnosed MM after 4 cycles of chemotherapy. However, this study lacks transplant correlation analysis due to small sample size and the tolerability of prolonged IRd treatment was unclear. We will continue to expand the sample size and extend follow‐up time to verify the conclusions of this study and uncover the tolerability of newly diagnosed MM patients to IRd.

## 5 CONCLUSION

In conclusion, this study retrospectively analyzed the efficacy, safety, PFS, and OS of different treatments in 52 newly diagnosed patients with MM, indicating the similar efficacy, PFS, and OS in patients treated by VRd and IRd. Moreover, IRd showed better safety than VRd, and it may be more convenient for patients with poor basic condition. Collectively, the present work indicated the efficacy and safety of IRd for newly diagnosed MM in the real‐world clinical setting, providing an insight for physicians to use IRd as first‐line treatment.

## AUTHOR CONTRIBUTIONS

Z. J. Huo, F. Chen, and Z. M. Luo designed this study. Z. J. Huo, F. Chen, and P. Liu collected and analyzed the data. Z. J. Huo and F. Chen searched the literature and drafted the manuscript. Z. M. Luo acquired the funding and revised the manuscript. All authors read and approved the final manuscript.

## FUNDING INFORMATION

The study was supported by China Hunan Provincial Science & technology Department (No. 2018SK52108).

## CONFLICT OF INTEREST

Authors have no potential conflict of interest to declare.

## ETHICS STATEMENT

This study was approved by the Medical Ethics Committee of Xiangtan Central Hospital (Approval number: 2019–01‐007).

## Data Availability

The data that support the findings of this study are available from the corresponding author upon reasonable request.
